# Nevirapine-Based Regimens in HIV-Infected Antiretroviral-Naive Patients: Systematic Review and Meta-Analysis of Randomized Controlled Trials

**DOI:** 10.1371/journal.pone.0076587

**Published:** 2013-10-07

**Authors:** Paweł Kawalec, Joanna Kryst, Alicja Mikrut, Andrzej Pilc

**Affiliations:** 1 Drug Management Department, Institute of Public Health, Faculty of Health Sciences, Jagiellonian University, Krakow, Poland; 2 Centrum HTA Sp. z o.o. Sp. komandytowa, Krakow, Poland; 3 Department of Neurobiology, Institute of Pharmacology, Polish Academy of Sciences, Krakow, Poland; Hopital Bichat Claude Bernard, France

## Abstract

**Background:**

Nevirapine belongs to the group of non-nucleoside reverse transcriptase inhibitors (NNRTIs) and is commonly administered in first-line treatment of HIV infection.

**Objective:**

Systematic review and meta-analysis was undertaken to compare effectiveness of nevirapine-based regimens with other antiretroviral schedules used as an initial treatment of HIV-infected antiretroviral-naive subjects.

**Methods:**

Electronic databases (PubMed, EMBASE, the Cochrane Library, Trip Database) were searched up to 28 December 2012 for randomized controlled trials (RCTs) published as a full text and regarding nevirapine-based regimens used as a initial treatment for HIV infection. Meta-analysis was performed with RevMan^®^ V 5.2 software.

**Results:**

Twelve RCTs were included in the systematic review and all of them were suitable for meta-analysis. Results of the meta-analysis have shown that nevirapine, efavirenz, and ritonavir-boosted protease inhibitor, added to the background regimens, were equally effective in terms of reaching undetectable plasma HIV RNA level as well as risk of disease progression or death. Compared with ritonavir-boosted protease inhibitor-based regimens, nevirapine-based regimens statistically significantly increased the risk of discontinuation of assigned treatment (RR=3.10; 95% CI: 1.14-8.41; p<0.05).

**Conclusions:**

Despite limited RCTs data available for particular comparisons, our results suggest that nevirapine-based regimens may be considered for first-line treatment of HIV-infected adults, due to their comparable efficacy to the other currently recommended initial antiretroviral therapies.

## Introduction

Although the efficacy of antiretroviral therapy in HIV-positive patients is indisputable, the variability of antiretroviral regimens used in clinical practice raises the question of the most effective treatment schedules. A combination of three or more drugs, known as highly active antiretroviral therapy (HAART), is now typically used. HAART is effective at lowering viral load and increasing CD4+ T cell levels [1]. According to the current practice guidelines antiretroviral regimens based on the combination of one non-nucleoside analogue reverse-transcriptase inhibitor (NNRTI) (commonly efavirenz or nevirapine) and two nucleoside analogue reverse transcriptase inhibitors (NRTIs) are among the preferred combinations for first-line antiretroviral therapy [2,3]. Such regimens have good virological potency and require administration once or twice-daily. Nevirapine, considered a “first-generation’’ NNRTI, has proven long term-efficacy and generally good tolerability in HIV-infected patients. Nevirapine is also used to prevent vertical transmission of HIV [4]. The new extended release formula of nevirapine facilitates therapy by reducing the number of pills to one a day [5]. Nevirapine-based regimens are preferred in resource-limited settings because of the lower cost in comparison with efavirenz, and the potential teratogenic effects of efavirenz. This is important, especially in African countries where the majority of antiretroviral treated adults are women and pregnancy rates in this population are high [6,7]. In light of numerous trials regarding the use of nevirapine in HIV-infected patients we systematically reviewed and meta-analyzed randomized controlled trial data in order to establish the differences between nevirapine-based regimens and other antiretroviral regimens used in HIV-infected patients not previously treated with antiretroviral therapy.

## Methods

This report was conducted according to the preferred reporting items for systematic reviews and meta-analyses (PRISMA) guidelines [8] and methods described in the Cochrane Handbook [9]. A systematic search of electronic databases and reference lists of all eligible studies published up to December 2012 was conducted to identify all relevant studies. The databases searched included Medline via PubMed, EMBASE, the Cochrane Central Register of Controlled Trials (CENTRAL), and the Trip Database. The search strategy included MeSH and EMTREE terms, combined with the Boolean logic operators AND and OR (Table 1). The Cochrane Database of Systematic Reviews, PubMed and EMBASE databases were also searched for review articles. The search results were restricted to human studies, and methodological filters were used for the selection of randomized controlled trials (RCTs). Studies were considered irrespective of language. We included all randomized controlled trials published as a full text comparing nevirapine with any other, commonly used treatment schedule in adult HIV-infected patients without prior exposure to antiretroviral therapy (studies assessing placebo as a comparator were excluded). Data presented only at conference meetings in abstract form were not included in the systematic review and meta-analysis, as the reliability of such results is lower than published peer-reviewed references. Studies including nevirapine administered to patients in every treatment arm, or given to pregnant or lactating women only for the prevention of mother-to-child transmission, and studies conducted only on children and infants were excluded. We also excluded studies conducted exclusively in HIV-infected patients with other concurrent infectious illnesses, such as hepatitis B, hepatitis C or tuberculosis. We searched for outcome measures assessing the clinical progression of disease or death, virological response (defined as undetectable plasma HIV RNA), and the safety profile (risk of adverse events and discontinuation of study because of adverse events).

**Table 1:** MeSH subject headings and EMTREE keywords used in search strategy construction (last updated: 28.12.2012).

Keywords (combined with boolean logical operators: AND, OR)	
Medical condition	(Viruses, Human Immunodeficiency) OR (AIDS Virus) OR (AIDS Viruses) OR (Virus, AIDS) OR (Viruses, AIDS) OR (HTLV-III) OR (Human Immunodeficiency Virus) OR (Human Immunodeficiency Viruses) OR (Human T Cell Lymphotropic Virus Type III) OR (Human T Lymphotropic Virus Type III) OR (Human T-Cell Leukemia Virus Type III) OR (Human T-Cell Leukemia Virus Type III) OR (Human T-Cell Lymphotropic Virus Type III) OR (Human T-Cell Lymphotropic Virus Type III) OR (Immunodeficiency Virus, Human) OR (Immunodeficiency Viruses, Human) OR (LAV-HTLV-III) OR (Lymphadenopathy-Associated Virus) OR (Lymphadenopathy-Associated Virus) OR (Lymphadenopathy-Associated Viruses) OR (Virus, Lymphadenopathy-Associated) OR (Viruses, Lymphadenopathy-Associated) OR (Virus, Human Immunodeficiency) OR (Acquired Immune Deficiency Syndrome Virus) OR (Acquired Immunodeficiency Syndrome Virus) OR (aids associated lentivirus) OR (aids associated retrovirus) OR (aids associated virus) OR (aids related virus) OR HIV OR (immunodeficiency associated virus) OR (immunodeficiency viruses primate) OR lav OR (LAV (AIDS)) OR (lentiviruses, primate) OR (lymphadenopathy associated retrovirus) OR (Lymphadenopathy associated virus) OR (virus, lymphadenopathy associated)
Intervention	(Nevirapine OR NVP OR Viramune OR (Promeco Brand of Nevirapine) OR (Cahill May Roberts Brand of Nevirapine) OR (BI-RG-587) OR (BI RG 587) OR BIRG587 OR birg 587 OR ciplanevimune OR nevimune OR viramun OR (viramune xr))
Methodological limits	PubMed: Humans, Randomized Controlled Trial; EMBASE: Humans, Randomized Controlled Trial, Embase only; CENTRAL: No limits applied; word variations have been searched

The search and selection of the trials were performed independently by two reviewers (P.K., J.K.). All disagreements were resolved by discussion with a third author (A.M.) to obtain consensus. Full texts of articles were reviewed according to the predefined inclusion or exclusion criteria. Data extracted by the first reviewer (P.K.) were verified by the second reviewer (J.K.). Extracted information included: study design, participant characteristics, interventions, duration of treatment, and clinical outcomes. To assess the methodological quality of included trials, the Jadad scale was used [10] (Table 2). Reduction of Risk Ratio (RR) was measured for data regarding the benefit of treatment, while for negative endpoints the increase of RR was assessed, all with 95% confidence intervals (CI). The results obtained from separate trials were combined using appropriate meta-analysis methods. An inverse variance and the Mantel-Haenszel or DerSimonian-Laird methods were used according to the data input and heterogeneity of test results. Clinical heterogeneity was assessed by examining the characteristics of the featured studies, whereas the statistical heterogeneity was assessed using the chi-square test, with a significance level of p<0.10. A fixed effects model was used when no statistical heterogeneity was detected; otherwise the random effects model was used. Meta-analysis was performed with RevMan^®^ V 5.2 software.

**Table 2:** Methodological quality of included RCTs.

Study (acronym if stated)	Jadad score						Allocation concealment
	1	2	3	4	5	Total	
Gaytán 2004 [15]	1	0	0	0	0	1	Not reported
Núñez 2002, SENC [16]	1	0	0	0	1	2	Not reported
van den Berg-Wolf 2008 [17], substudy of FIRST [18]	1	0	0	0	0	1	Not reported
van Leth 2004, 2NN [19]	1	0	0	0	1	2	Described
Wester 2010, TSHEPO [20]	1	0	0	0	1	2	Not reported
Dejesus 2011, NEWART [21]	1	1	0	0	1	3	Described
Harris 2009 [22]	1	0	0	0	0	1	Not reported
Lapadula 2008 [23]	1	0	0	0	1	2	Not reported
Lockman 2010, OCTANE [24]	1	0	0	0	1	2	Not reported
Lockman 2012, OCTANE [25]	1	0	0	0	1	2	Described
Lowe 2005, ARES [26]	1	0	0	0	1	2	Not reported
Soriano 2011, ARTEN [27]	1	0	0	0	1	2	Not reported

1 - Was the study described as randomized?; 2 - Was the method of randomization described and appropriate?; 3 - Was the study described as double blind?; 4 - Was the method of blinding described and appropriate?; 5 - Were withdrawals and dropouts described?

## Results

**Figure 1:**
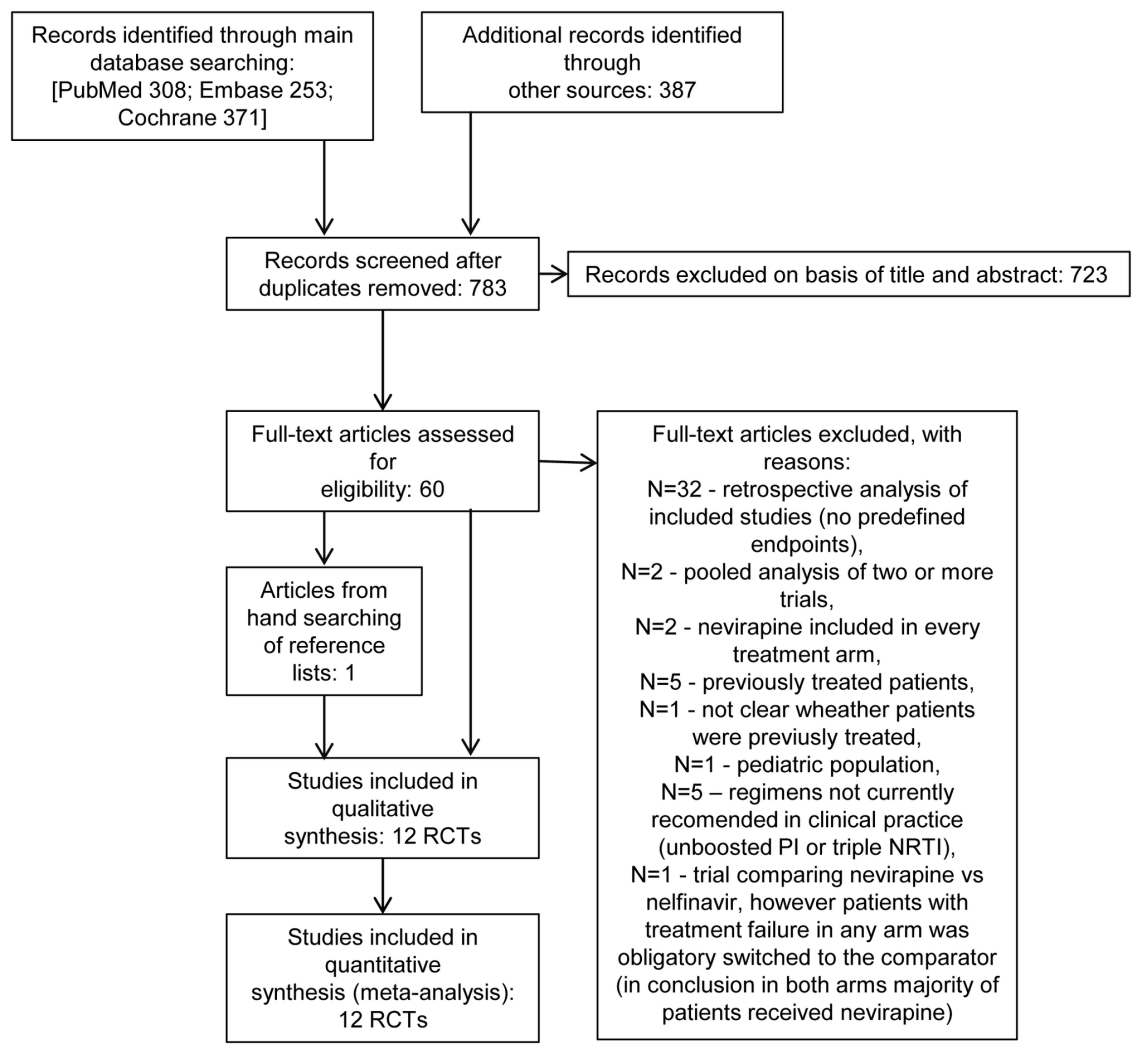
PRISMA flow diagram for selection of studies identified in the systematic review.

The electronic search yielded 783 items after duplicates were removed. The selection of titles and abstracts resulted in 60 potentially relevant full-text articles, of which 49 references were excluded due to the reasons presented in Figure 1. Twelve studies met the predefined inclusion criteria for systematic review and were suitable for quantitative synthesis (meta-analysis). The flow of selection through the different phases of the systematic review is shown in Figure 1.

Twelve randomized controlled trials published in English as peer-reviewed articles were included. These included studies were grouped in the following way. Firstly, background regimen, common in compared groups, was identified. Active drugs added to the common background regimen were considered as comparators. Two different regimens adequate for comparisons with nevirapine were identified: 1 NNRTI or bPI (ritonavir-boosted protease inhibitor), all the above added to specified background regimens. We did not take into consideration regimens containing unboosted PI and triple NRTIs regimens as they are no longer used in clinical practice.

A predefined inclusion criterion for studies was the absence of any prior treatment with antiretroviral therapy. Finally, trials recruiting patients with limited previous exposure to antiretroviral therapy were included (Table 3). Data of clinical relevant endpoints reflecting advances in the treatment of HIV infection were extracted from the studies. Disease progression was evaluated according to the Centers for Disease Control (CDC) classification [11] or WHO guidelines, although definitions of this endpoint were not consistent across the included studies. Disease progression was determined by the occurrence of clinical features indicating a higher CDC/WHO stage (most often: new C or B/C events or new 3/4 AIDS-defining events) and predominately, but not always, the occurrence of death.

**Table 3:** Characteristics of the randomized controlled trials for nevirapine compared to different regimens used to treat antiretroviral–naive HIV-infected adult patients.

Study author, year of publication, study type, sites	Population	Study duration	Interventions&	Study outcomes#
background regimen (2 NRTIs/2NRTIs+1PI) + nevirapine vs background regimen + 1NNRTI				
Gaytán 2004 [15], RCT, open-label, 1 center in Mexico	ART-naive, age ≥18 years, pVL >55 000 copies/ml, N=58	48 weeks	A: AZT + 3TC + NVP, N=28 B: AZT + 3TC + EFV, N=30	disease progression or death, percentage of patients with pVL <400 copies/ml (week 48), discontinuation of therapy due to adverse events
Núñez 2002, SENC [16], RCT, open-label, 1 center in Spain	ART-naive, age > 18 years, pVL: 500-100 000 copies/ml, N=67	48 weeks	A: ddI + d4T + NVP, N=36B: ddI + d4T + EFV, N=31	percentage of patients with pVL <50 copies/ml (week 48), discontinuation of therapy due to adverse events
van den Berg-Wolf 2008, NNRTI [17] substudy of FIRST trial [18], RCT, open-label, 17 clinical trials units at 80 sites in the United States	ART-naive (less than 4 weeks of prior NRTI use or 1 week of 3TC use was allowed), age ≥13 years, N=228	median -5 years	patients randomized to NNRTI+NRTIs strategy (N=110) or PI+NNRTI+NRTIs strategy (118) and then randomized to: A: EFV, N=111; B: NVP, N=117 (NRTIs included: ABC + 3TC or ddI + d4T or AZT + 3TC or d4T + 3TC; PI included: NFV, INV, /r	disease progression or death, percentage of patients with pVL <50 copies/ml (week 52), discontinuation of therapy due to adverse events
van Leth 2004, 2NN [19], RCT, open-label, centers in North and South America, Australia, Europe, South Africa and Thailand	ART-naive, age ≥16 years, pVL >5 000 copies/ml, N=1216	48 weeks	A: d4T + 3TC + NVP (400 mg once daily), N=220; B: d4T + 3TC + NVP (200 mg twice daily), N=387; C: d4T+ 3TC+ EFV (600 mg once daily), N=400	disease progression or death, percentage of patients with pVL <50 copies/ml (week 48), discontinuation of therapy due to adverse events
Wester 2010, TSHEPO [20], RCT, open-label, one center in Botswana	ART-naive, age ≥ 18 years, pVL >55 000 copies/ml, N=650	3 years	A: 2NRTI + NVP, N=325; B: 2NRTI + EFV, N=325 (2 NRTI consisted: AZT + 3TC or AZT + ddI or d4T + 3TC)	percentage of patients with pVL <400 copies/ml (week 52), in safety analysis treatment-modifying toxicities were evaluated (not suitable for meta-analysis)
background regimen (2 NRTIs) + nevirapine vs background regimen + bPI				
Dejesus 2011, NEWART [21], RCT, open-label, 18 study sites in United States	ART-naive (up to 10 days of prior NRTIs or NNRTIs, all other classes of antiretroviral agents was allowed up to 2 weeks), CD4 <400 cells mm^3^ (men), <250 cells mm^3^ (women), N=154	48 weeks	A: TDF + FTC + NVP, N=76; B: TDF + FTC + ATV/r, N=78 (2 patients were excluded from the analyses due to methodological error)	proportion of patients with pVL <50 copies/ml (week 48), discontinuation of therapy due to adverse events
Harris 2009 [22], RCT, open-label, centers in Canada, France, Spain, Argentina	ART-naive, age ≥ 18 years, pVL >5 000 copies/ml, N=77	96 weeks	A: AZT + 3TC + NVP, N=26; B: AZT + 3TC + LPV/r, N=25	percentage of patients with pVL<50 ml (week 48 and 96), discontinuation of therapy due to adverse events
Lapadula 2008 [23] RCT, open-label, 1 center in Italy@	ART-naive, age >18 years, CD4 cell count <400 cells/mm^3^ (men), <250 cells/mm^3^ (women), N=14	12 weeks	A: TDF + FTC + NVP, N=7; B: TDF + FTC + ATV/r, N=7	efficacy data reported in study were not suitable for meta-analysis, discontinuation of therapy due to adverse events
Lockman 2010, OCTANE [24], RCT, open label, centers in Botswana, Kenya, Malawi, South Africa, Uganda, Zambia, and Zimbabwe	ART-naive (all participants had received single-dose of NVP≥6 months before enrollment, up to 10 weeks of prior AZT was allowed), CD4 <200 cells/mm^3^, N=243	≥48 weeks	A: TDF + FTC + NVP, N=123; B: TDF + FTC + LPV/r, N=120 (2 women did not received study medication and were excluded from the analyses)	disease progression or death, in the efficacy outcomes virological failure was defined as a composite endpoint (not suitable for meta-analysis), risk of grade 3 or higher adverse events, discontinuation of therapy due to adverse events
Lockman 2012, OCTANE [25], RCT, open label, centers in Botswana, Kenya, Malawi, South Africa, Uganda, Zambia, and Zimbabwe	ART-naive (up to 10 week of prior AZT was allowed), women only, CD4 <200 cells/mm^3^, N=502	≥48 weeks	A: TDF + FTC + NVP, N=251; B: TDF + FTC + LPV/r, N=251 (2 women did not received study medication and were excluded from the analyses)	disease progression or death, virological failure was defined as a composite endpoint (not suitable for meta-analysis), risk of grade 3/4 adverse events, discontinuation of therapy due to adverse events
Lowe 2005, ARES [26], RCT, open-label, 7 centers in Netherlands	ART-naive, age ≥18 years, pVL ≥5 000 copies/ml, N=71	48 weeks	B: ddI + 3TC + NVP, N=22; C: ddI + 3TC + SQV/r, N=23	disease progression or death, percentage of patients with pVL <50 copies/ml (week 48), risk of grade 3/4 adverse events, discontinuation of therapy due to adverse events
Soriano 2011, ARTEN [27], RCT, open-label, 68 centers in Argentina, Germany, Italy, Mexico, Poland, Romania, Spain, Switzerland and the UK	ART-naive (up to 7 days of prior treatment was allowed), age ≥18 years, CD4 <400 cells/mm^3^ (men), <250 cells/mm^3^ (women), N=576	48 weeks	A: TDF + FTC + NVP 200 mg twice daily, N=192; B: TDF + FTC + NVP 400 mg once daily, N=191; C: TDF + FTC + ATV/r, N=193 (7 patients did not received study medication and were excluded from the analyses)	percentage of patients with pVL <50 copies/ml (week 48), discontinuation of therapy due to adverse events

3TC - lamivudine, ABC - abacavir, ART – antiretrovital therapy, ATV - atazanavir, AZT - zidovudine, d4T - stavudine, ddI - didanosine, EFV - efavirenz, FTC - emtricitabine, NFV - nelfinavir, NNRTI - non-nucleoside reverse transcriptase inhibitor, NRTI - nucleoside reverse transcriptase inhibitor, NVP - nevirapine, PI - protease inhibitor, /r - low-dose ritonavir, SQV - saquinavir, TDF - tenofovir, pVL - plasma HIV RNA.

# study outcomes included in meta-analysis only.

& − interventions included in meta-analysis only.

@ prematurely stopped due to virological failure.

Plasma viral load (pVL) is a globally accepted endpoint used to measure the efficacy of antiretroviral drugs [12]. Available data of virological responses in the included studies were reported as plasma HIV RNA level below: 50 and 400 copies/ml. It should be noted that the suppression of pVL to 50 copies/ml is a better predictor for durable virological success than a suppression to <400-500 copies/ml [13,14]. Data for nevirapine administered in different doses in one study were aggregated. For safety analysis an overall risk of grade 3/4 adverse events was assessed when it was possible; otherwise clinical adverse events data were included.

The characteristics of studies included in this report are presented in Table 3. All of studies included in the systematic review were open-label. Three of the included trials provided information about allocation concealment. Most of the trials included data about patient withdrawal and drop-out from the study. Jadad scores ranged from 1 to 3, mostly due to a lack of blinding and insufficient details about randomization methods used (Table 2).

### Effectiveness of adding nevirapine vs one non-nucleoside reverse transcriptase inhibitor (NNRTI) to the background regimen

**Figure 2:**
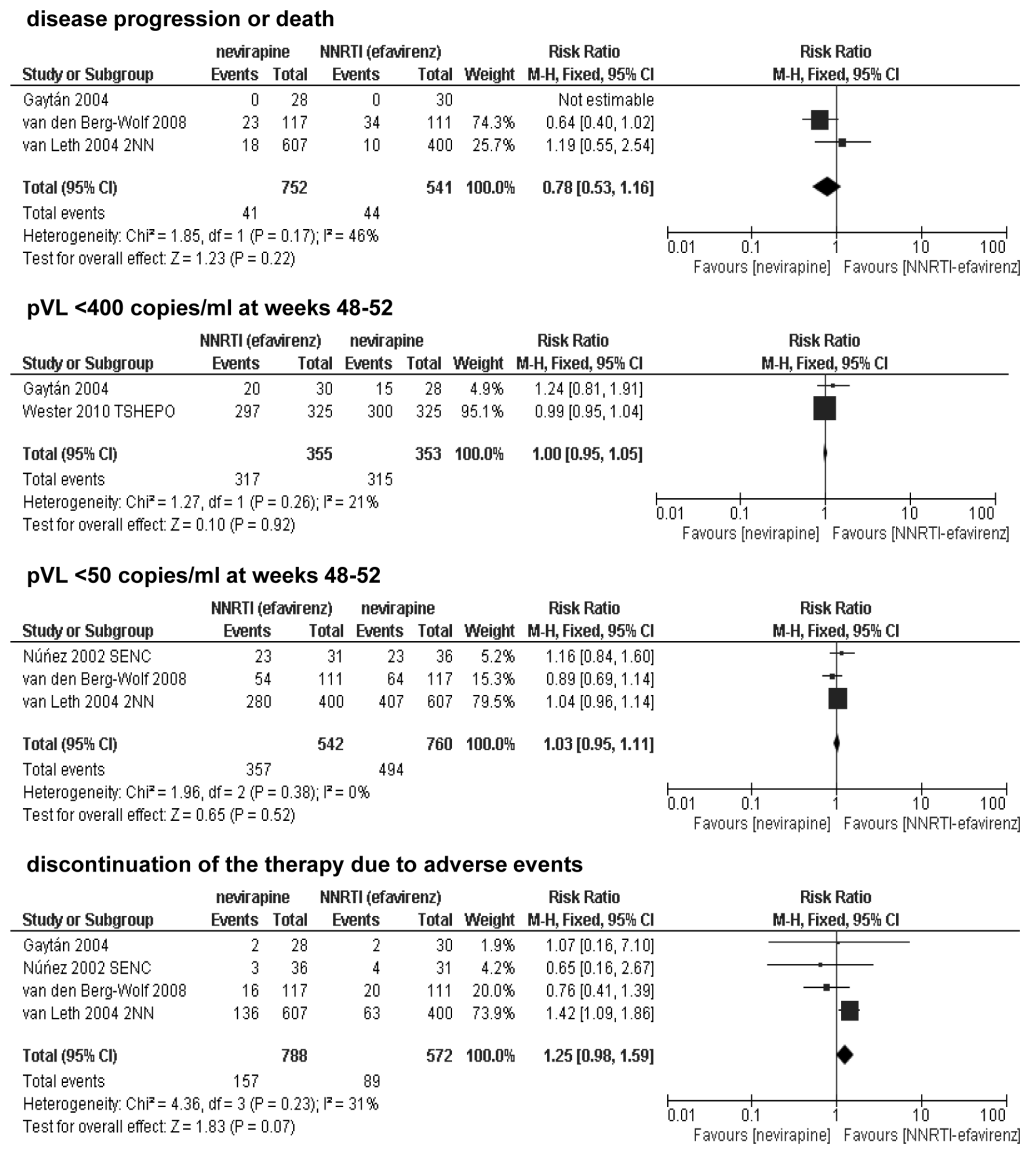
Forest plot of comparison: nevirapine vs 1 NNRTI (efavirenz) added to the background regimen.

All studies [15,16,17,18,19,20] suitable for inclusion for the comparison of nevirapine vs one NNRTI evaluated efavirenz as NNRTI. This means that all comparisons were performed between nevirapine and efavirenz. The FIRST substudy [17] included patients with previous limited exposure to antiretroviral therapy. Three studies evaluated patients during the course of 48 weeks, and in the other two trials the follow-up period lasted 3 to 5 years (median). Studies were heterogeneous regarding baseline plasma HIV RNA level (>500 to >55 000 copies/ml). Data for nevirapine in different doses in two arms in the 2NN [19] study were aggregated. The differences between nevirapine and efavirenz were not statistically significant for the proportion of patients with disease progression or death (RR=0.78; 95% CI: 0.53-1.16; p>0.05), nor for the proportion of patients with virological response (plasma viral loads below 400 copies/ml: RR=1.00; 95% CI: 0.95-1.05; p>0.05 and below 50 copies/ml: RR=1.03; 95% CI: 0.95-1.11; p>0.05) in weeks 48-52. The results of meta-analysis showed that the risk of assigned treatment discontinuation due to intolerance was comparable in both arms (RR=1.25; 95% CI: 0.99-1.60; p>0.05); Figure 2.

### Effectiveness of adding nevirapine vs ritonavir-boosted protease inhibitor (bPI) to the background regimen

**Figure 3:**
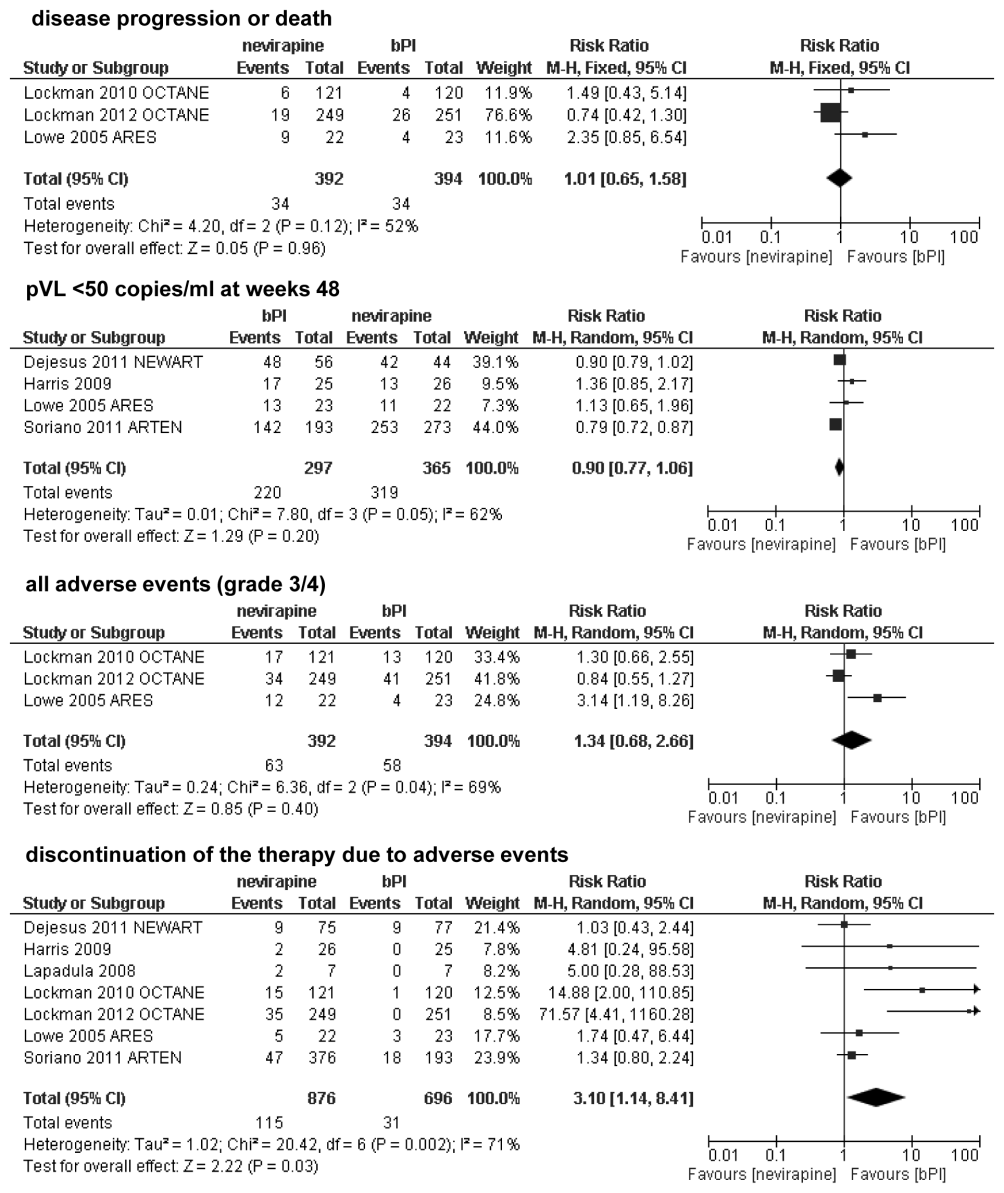
Forest plot of comparison: nevirapine vs bPI added to the background regimen.

Of seven studies ( [21,22,23,24,25,26,27]) comparing nevirapine vs ritonavir-boosted protease inhibitor (bPI) only three trials included truly antiretroviral naive patients; four other studies recruited patients with limited prior antiretroviral exposure. All patients in the OCTANE 2010 [24] trial had received a single-dose of nevirapine six or more months before enrollment, for the prevention of mother-to-child HIV transmission. In most studies results for a 48-week follow-up period were reported. Additionally, one study [23] lasting only 12 weeks was prematurely discontinued due to virological failure, and as such its efficacy results seemed to be unsuitable for the meta-analysis. In the ARES [26] trial, data presented for disease progression were defined as the occurrence of new CDC events only (B/C events), and there was no information about deaths in the whole article (of note: in the OCTANE study disease progression was defined as the occurrence of a new CDC event or death). No statistically significant differences between groups were observed in regards to the risk of disease progression or death (RR=1.01; 95% CI: 0.65-1.58; p>0.05), or proportions of patients with plasma viral loads <50 copies/ml at week 48 (RR=0.90; 95% CI: 0.77-1.06; p>0.05). While there were no statistically significant differences between analyzed regimens with respect to incidences of adverse events of grade 3/4 (RR=1.34; 95% CI: 0.68-2.66; p>0.05), risk of treatment discontinuation due to adverse events was statistically significantly higher in the nevirapine group compared to the 2 PI-based regimen (RR=3.10; 95% CI: 1.14-8.41); Figure 3.

## Discussion

To our knowledge this is the first broad systematic review containing meta-analysis that compares nevirapine-based therapy with other antiretroviral regimens used to treat antiretroviral-naive HIV-infected adult patients. The overall results of the performed meta-analysis show that the efficacy of nevirapine is comparable to efavirenz and ritonavir-boosted protease inhibitors when added to the background regimen (usually 2 NRTIs) in the target population of treatment-naive adult HIV-infected patients. These results are supported by clinical guidelines that recommend the above regimens as initial therapeutic options for HIV infection. Our results are also consistent with previous meta-analyses, showing that nevirapine and efavirenz in combination with 2 NRTIs given to antiretroviral-naive HIV-infected patients have similar efficacy [28]. It should be noted that we confirm the efficacy results of Mbuagbaw et al. [28] despite excluding patients with concomitant tuberculosis and data from meeting abstracts. The reasons for excluding patients with concurrent HIV infection and tuberculosis are based on the WHO guidelines that recommend treatment with efavirenz but not nevirapine in individuals co-infected with tuberculosis and HIV who are receiving rifampicin-based therapy [29]. The results obtained in the presented meta-analysis also confirm previous findings where combined data from two studies showed similar efficacy of nevirapine versus efavirenz in combination with stavudine and lamivudine (2 NRTIs) for first-line treatment of HIV infection [30].

The main limitation of the conducted meta-analysis was the number of trials available for particular comparisons and heterogeneity of the included studies regarding background regimens, baseline characteristics of randomized patients (especially proportions of patients in various stages of disease), lengths of follow-up periods, and differences in the analyzed endpoint definitions. Some of the included studies recruited patients with previous limited exposure to antiretroviral therapy, for example single exposure to nevirapine for the prevention of mother-to-child transmission of HIV. Nevirapine, as well as other NNRTIs, has a low genetic barrier. The OCTANE study showed that resistance to nevirapine occurred in 14% of women with previous exposure to a single-dose of nevirapine, leading to decreased efficacy of subsequent nevirapine-based treatments in comparison to NNRTI-naive women [24,25]. However, excluding data from the OCTANE 2010 [24] study did not changed the results of our meta-analysis regarding efficacy (data not shown). The heterogeneity of the included studies also regarded baseline HIV RNA plasma levels, which was shown previously as a predictive factor of reaching virological response to treatment [25]. The results of meta-analysis performed by Raboud et al. showed that patients with a baseline pVL <100 000 copies/ml were more likely to achieve virological response (pVL <400 copies/ml and <20 copies/ml) than those with baseline plasma HIV RNA level >100 000 copies/ml [31]. Guidelines for the treatment of HIV-infected adults indicate low baseline viremia as a predictor of virologic success during antiretroviral therapy [3].

Moreover, in some studies the exact data on the particular, clinically relevant outcomes were unavailable within the papers, or they were not intended to be evaluated at all. Therefore, only some of the identified studies could be included in a quantitative analysis, and only some of the presented results were suitable for meta-analysis, especially in the case of efficacy evaluation (see Table 3).

Another limitation of the performed meta-analysis is the absence of subgroup analysis according to background regimens, due to the limited number of trials adequate for inclusion in separate comparisons. Bartlett et al. [32] compared different background regimens containing 2 NRTIs in efavirenz-based therapy. Among the most effective NRTI regimens were combinations of tenofovir and emtricitabine, while the less effective regimens contain zidovudine and lamivudine (about 10% difference in the proportion of patients with the time to loss of virologic response at week 48) [32]. The combination of tenofovir plus emtricitabine is also among the most preferred background regimens recommended in the current practice guidelines [2,3]. However, in our meta-analysis the combination of tenofovir and emtricitabine was only used as a background regimen in trials comparing nevirapine and bPI-based therapy.

We did not perform subgroup analysis according to nevirapine dosage. Most of the included studies analyzed nevirapine given according to product characteristic -200 mg twice a day. Three trials used nevirapine given as one daily dose of 400 mg [19,20,24]. Nevertheless, results of the 2NN study [19] showed similar rates of treatment failure when nevirapine was given either once or twice daily.

It could be suggested that a network meta-analysis to support the results obtained in direct analyses should be performed. However, a mixed treatment comparison (MTC), a special case of network meta-analysis combining direct evidence and indirect evidence for particular pairwise comparisons, has several limitations. First, the mixed treatment approach is not a substitute for a large, well-designed randomized controlled trial examining relative clinical efficacy and safety. The validity of the adjusted indirect comparisons (as with MTC) depends on the internal validity and homogeneity of the included trials [33,34,35]. The mixed treatment approach, while efficient in deriving treatment estimates, is only as good as the data that are included. The differences in study inclusion criteria meant disparity in the patient populations between the trials. Unfortunately, relevant clinical heterogeneity of the studies included in the performed meta-analysis was found (as mentioned above, referring for example to various agents from a particular class of antiretroviral drugs used in a background therapy and different stages of AIDS or baseline plasma HIV RNA levels in patients participating in the included studies). In that situation the strength of results from a mixed-treated comparison could be limited. Moreover, Chou et al. provided evidence for a strong discrepancy between direct and indirect comparisons in an HIV-infected population. Crucially, while direct meta-analysis showed that NNRTI-based regimens are better than PI-based regimens (in terms of the virological response), an indirect comparison revealed opposite results. Chou and colleagues conclude that indirect comparisons within the mixed treatment comparisons may not be reliable for such complex treatments as HAART, especially in the case of paucity of data or imbalance of available data concerning the evaluated drugs, or significant heterogeneity among them [36].

According to the practice guidelines antiretroviral therapy in HIV-infected patients should maximally and durably suppress plasma HIV viral load and also reduce HIV-associated morbidity and prolong the duration and quality of survival [3]. In our meta-analysis the clinical endpoint, defined as disease progression and/or death, showed no statistical difference between nevirapine and efevirenz, nor between nevirapine and ritonavir-boosted protease inhibitor (bPI) therapy. However, it should be noted that not all of the included studies provided data regarding the above endpoint (probably due to the short follow-up time). What is more, the low rate of disease progression or death reported in the included trials hampers statistical analysis. However, results from long-term studies assessing disease progression or death as a primary endpoint could verify the results obtained in the performed meta-analysis. The results obtained in a group of Senegalese patients followed-up for a median of 48 months showed no differences between nevirapine and efavirenz with respect to the time to death or time to AIDS progression [37].

Results from other observational studies do not give a clear answer on the issue of the higher efficacy of nevirapine or efavirenz in the analyzed population. The above-mentioned study conducted in Senegalese patients [37], as well as the results of Patel et al. trial [38], showed comparable efficacy of nevirapine and efavirenz in antiretroviral treatment-naive patients. On the other hand, a prospective, observational trial involving more than 20 000 patients showed a lower incidence of death and AIDS-defining illness, as well as lower risk of virologic failure at 12 months for efavirenz compared with nevirapine [39]. Results obtained in routine clinical practice in Europe also showed that patients starting efavirenz had a 48% lower risk of discontinuation due to treatment failure compared with the nevirapine group (however, in this trial about 60% of patients were treatment-experienced) [40].

The overall toxicity profiles of nevirapine and other regimens were comparable. However, a higher risk of treatment discontinuation due to adverse events in comparison to ritonavir-boosted protease inhibitor (bPI) was found. Due to limited data it was also not possible to compare particular adverse events between analyzed regimens. Regarding the safety profile of nevirapine, hepatotoxicity and skin reactions, especially rashes, are a special concern. In a large prospective controlled trial that included nevirapine as a part of the antiretroviral regimen, a high frequency of hepatotoxicity was observed [41]. Data from the Asian observational database on HIV treatment showed that the risk of discontinuation of nevirapine therapy due to rashes and hepatotoxicity were 7% and 2%, respectively [42]. Since the greatest risk of hepatotoxicity and rashes occurs in the first 6 weeks of therapy [43], patients should be closely monitored during the initiation of nevirapine therapy. Among the specific factors, gender and pretreatment CD4 count should be considered when antiretroviral nevirapine-based therapy is initiated [3]. The above factors are associated with the greater risk of hepatic adverse events [43].

Results of previous meta-analyses comparing nevirapine and efavirenz in combination with other antiretroviral agents showed differences between both drugs in terms of toxicity [28,30]. Siegfried et al. [30] showed a higher frequency of toxicity in participants receiving nevirapine compared to those treated with efavirenz. However, the authors included only 2 trials where NNRTIs were given in combination with stavudine and lamivudine [30]. Mbuagbaw et al. [28] performed a comparison regarding particular adverse events reported in nevirapine- and efavirenz-based regimens in antiretroviral-naive HIV-infected patients. Efavirenz was connected with a higher risk of central nervous system side-effects, while more patients with raised transaminases and neutropenia were reported in nevirapine arms [28]. In our meta-analysis discontinuation of treatment due to adverse events showed a tendency to favour efavirenz, although the observed effect was not statistically significant. It should be noted that due to the absence of central nervous system adverse events and limited influence on lipid profiles, nevirapine is a safe therapeutic option for patients at risk of depression or cardiovascular disease [44].

In summary, our data demonstrate the comparable efficacy of nevirapine-based therapy versus other regimens recommended as initial therapy for HIV-infected patients (PI-based and efavirenz-based treatments). Concerning safety, special groups of patients can achieve significant clinical benefits from nevirapine-based regimens. However, when nevirapine treatment is initiated the risk of hepatotoxicity and rashes should be taken into account. Despite the potential limitations of the performed meta-analysis our results provide important guidance for choosing first-line nevirapine-based treatment for HIV-infected patients.

## Supporting Information

Checklist S1 **PRISMA Checklist.**
(DOC)
